# First metagenomic analysis of the Andean condor (*Vultur gryphus*) gut microbiome reveals microbial diversity and wide resistome

**DOI:** 10.7717/peerj.15235

**Published:** 2023-07-07

**Authors:** J. Eduardo Martinez-Hernandez, Pablo Berrios, Rodrigo Santibáñez, Yesid Cuesta Astroz, Carolina Sanchez, Alberto J. M. Martin, Annette N. Trombert

**Affiliations:** 1 Laboratorio de Redes Biológicas, Centro Científico y Tecnológico de Excelencia Ciencia & Vida, Fundación Ciencia & Vida, Santiago, Chile; 2 CGNA (Agriaquaculture Nutritional Genomic Center), Temuco, Chile; 3 Escuela de Biotecnología, Facultad de Ciencias, Ingeniería y Tecnología, Universidad Mayor, Santiago, Región Metropolitana, Chile; 4 Instituto Colombiano de Medicina Tropical, Universidad CES, Sabaneta, Colombia; 5 Centro de Oncología de Precisión, Escuela de Medicina, Universidad Mayor, Santiago, Chile; 6 Advanced Genomics Core, Universidad Mayor, Santiago, Chile; 7 Escuela de Ingeniería, Facultad de Ingeniería, Arquitectura y Diseño, Universidad San Sebastián, Santiago, Chile

**Keywords:** Andean condor, Metagenomics, Gut microbiome, *Clostridium perfringens*, Virulome, Resistome, Antibiotic resistance

## Abstract

**Background:**

The Andean condor (*Vultur gryphus*) is the largest scavenger in South America. This predatory bird plays a crucial role in their ecological niche by removing carcasses. We report the first metagenomic analysis of the Andean condor gut microbiome.

**Methods:**

This work analyzed shotgun metagenomics data from a mixture of fifteen captive Chilean Andean condors. To filter eukaryote contamination, we employed BWA-MEM v0.7. Taxonomy assignment was performed using Kraken2 and MetaPhlAn v2.0 and all filtered reads were assembled using IDBA-UD v1.1.3. The two most abundant species were used to perform a genome reference-guided assembly using MetaCompass. Finally, we performed a gene prediction using Prodigal and each gene predicted was functionally annotated. InterproScan v5.31-70.0 was additionally used to detect homology based on protein domains and KEGG mapper software for reconstructing metabolic pathways.

**Results:**

Our results demonstrate concordance with the other gut microbiome data from New World vultures. In the Andean condor, Firmicutes was the most abundant phylum present, with *Clostridium perfringens*, a potentially pathogenic bacterium for other animals, as dominating species in the gut microbiome. We assembled all reads corresponding to the top two species found in the condor gut microbiome, finding between 94% to 98% of completeness for *Clostridium perfringens* and *Plesiomonas shigelloides*, respectively. Our work highlights the ability of the Andean condor to act as an environmental reservoir and potential vector for critical priority pathogens which contain relevant genetic elements. Among these genetic elements, we found 71 antimicrobial resistance genes and 1,786 virulence factors that we associated with several adaptation processes.

## Introduction

Vultures are obligate scavengers, birds of prey that provide several ecological services such as nutrient recycling or avoiding soil contamination by carcass feeding, which help to reduce the spread of diseases in their habitats (Ogada, Keesing & Virani, 2012; Chung et al., 2015; Buechley & Şekercioğlu, 2016). Vultures are classified into two non-phylogenetically related groups (Wink, 1995; Johnson et al., 2016): Old World vultures inhabiting Africa, Asia, and Europe that find carcasses exclusively by sight, and New World vultures, located in North and South America that find carcasses by sight and by smell (Seibold & Helbig, 1995).

Previous studies have shown the ability of vultures to tolerate feeding on carcasses containing a wide range of pathogenic microorganisms. A study performed by Roggenbuck et al. (2014) showed a low diversity of species in the hindgut of New World vultures determined by the acidity of the hindgut and microbial competition for the available nutrients. Also, in this work, the microbiome of the New World vultures was identified. Specimens of the black vulture (*Coragyps atratus*) and the turkey vulture (*Cathartes aura*) were used for facial skin and guts microbiome analyses, finding that the gut microbiota of these vultures is dominated by the genera *Clostridia* and *Fusobacteria* (Roggenbuck et al., 2014). Zepeda Mendoza et al. (2018) showed that the most abundant bacterial genus in the facial skin microbiome was *Pseudomonas, Bacteroides*, and *Prevotella*. In contrast, *Escherichia, Campylobacter*, and *Clostridium* were the most abundant genus in the gut microbiomes of black and turkey vultures. Meng et al. (2017) and co-workers aimed to characterize the gut microbiome of Old World vultures, identifying that *Clostridium perfringens* is the most abundant species present in the gut of Old World vultures. Another study by Blanco et al. (2020) has also linked the diet of Old World vultures, usually dominated by domestic animals, as the source of frequent genes involved in the resistance to antimicrobial agents in the gut microbiome. These results coincide with studies performed with New World vultures (Roggenbuck et al., 2014; Zepeda Mendoza et al., 2018) and demonstrate that vultures are reservoirs for bacteria that may be pathogenic to other animals. In addition, these findings also indicate adaptive processes in vultures and the possible protective role of other species of the *Clostridium* genus, such as *C. carboxidovorans*, *C. sporogenes*, and *C. butyricum* (Meng et al., 2017; Zepeda Mendoza et al., 2018). However, the diversity of Andean condor microbiota is still unknown.

Condor, derived from the Quechua *Kuntur*, is the common name for two species of New World vultures: the Andean condor (*Vultur gryphus*) and the California condor (*Gymnogyps californianus*). The Andean condor is the largest scavenger in South America and is distributed from Northern Colombia through the Andes mountains to Tierra del Fuego in Argentina and Chile (Naveda-Rodríguez et al., 2016). Near 50 percent of the population inhabits Chile and Argentina (Naveda-Rodríguez et al., 2016). According to The International Union for Conservation of Nature Red (IUNC) list (IUCN, 2022), the Andean condor is considered “Near Threatened” (NT). Thus, it is a species with a population that is suspected to be declining moderately rapidly owing to persecution by man (Méndez et al., 2015). Specifically, the population that inhabits the central valley of Chile is one of the most threatened due to the modification of habitat and food sources by anthropogenic activities (Duclos et al., 2020; Méndez et al., 2021).

The Andean condor, like other vultures, plays an important ecological role as a biodegradator by nutrient recycling and removal of decomposing organic material thereby preventing the spread of infectious diseases associated with the rotting of cadavers (Ogada, Keesing & Virani, 2012; Buechley & Şekercioğlu, 2016; García-Alfonso et al., 2019). In nature, the Andean condor consumes carcasses from guanacos (*Lama guanicoe*), rheas, goats, sheep, hares, rabbits, cows, and horses (Lambertucci et al., 2009). On the other hand, it has been recorded that the diet of the Andean condor also may consist of marine animals such as whales, sea lions, penguins, or even pelicans (Lambertucci et al., 2018). The diet composition of condors could impact the entire local population and have a decisive impact on scavengers’ survival in the wild and in captivity (Lambertucci et al., 2009; Gaengler & Clum, 2015).

Metagenomics studies contribute to producing microbial draft genomes from new biomes to understand the impact on animal/human health and biodiversity conservation (Gilbert & Dupont, 2011; Martín et al., 2014; Mendes et al., 2017). From a metagenomic approach, the Andean condor microbiome remains largely unexplored. In this sense, this biome has a huge gap in metagenomics information. It is necessary to include this kind of information to have a global picture of the diversity of microorganisms and their relevant features, mainly virulence and antimicrobial resistance.

Based on metagenomics data from this biome is possible to study virulence factors since vultures are exposed to various pathogens, including those that cause anthrax, tuberculosis, and brucellosis (Zepeda Mendoza et al., 2018). Through the study of virulence factors is possible to characterize these infections and propose potential biomarkers for the diagnosis of etiological agents of infectious diseases, and this is directly related to their biology and the susceptibility to the pathogens in their diet and the role in the transmission of infectious diseases (Zepeda Mendoza et al., 2018).

On the other hand, the analysis of antimicrobial-resistant (AMR) genes in our study is relevant since the spread of AMR bacteria is one of the greatest threats to human medicine, veterinary medicine, and public health (Laxminarayan et al., 2013) and the role of wild animal species in the maintenance and transmission of these resistance genes is still poorly understood.

We analyzed shotgun metagenomics data from captive Chilean Andean condor specimens in this work. We focused on the Andean condor gut microbiota to discover and understand the abundance patterns of microorganisms associated with this unique and interesting biome and characterize genes related to virulence and antimicrobial resistance in this microbial community. Specifically, we will focus on virulence and antibiotic resistance genes due to the potential implications of these as therapeutic targets to treat bacteria-caused diseases in other species, such as human and domestic animals.

## Materials and Methods

### Sampling method and DNA sequencing

Fresh stool samples of 15 captive condors with the same diet conditions were collected on June 2017 from Centro de Rehabilitación de Aves Rapaces (CRAR) located in Talagante, Metropolitan Region, Chile (33°41′01.5″S, 70°54′23.4″W). All animals shared the same cage and stools were collected immediately after deposition during daily cage cleaning activities, avoiding any interaction with the birds. Samples were recovered in 15 mL sterile centrifuge Falcon tubes, avoiding soil particles. All samples were transported in ice before storage at −20 °C until further use. Due to the collection method, no ethical approval was required.

Frozen stool samples were processed together and mixed into a single sample. Bacterial DNA was extracted using QIAamp DNA Stool Mini Kit (Qiagen, Hilden, Germany) following the manufacturer’s instructions and a DNA pool was obtained. DNA concentration was evaluated using QubitTM ds DNA HS Assay Kit (Thermo Fisher Scientific, Waltham, MA, USA) and integrity was analyzed by agarose (0.8%) electrophoresis in TAE (Tris-Acetate-EDTA) buffer. A total of 100 ng of DNA was randomly fragmented using enzymatic fragmentation with NEBNext® dsDNA Fragmentase® (New England Biolabs, Ipswich, MA, USA) and used to construct a DNA library with the Illumina TruSeq DNA Nano Sample Preparation Kit (Illumina, San Diego, CA, USA). The quality and size distribution of the library was evaluated with the Agilent 2100 Bioanalyzer using a DNA 1000 chip (Agilent Technologies, Santa Clara, CA, USA) and was quantified using the KAPA Library Quantification Kit for Illumina Platforms (Kapa Biosystems, Wilmington, MA, USA) on the Step One Plus Real-Time PCR System (Applied Biosystems, Waltham, MA, USA). The library was sequenced using the HiSeq 2500 platform with paired-end sequencing (2 × 100 bp).

### Metagenomic assembly and taxonomical annotation

Raw reads quality control was performed using FastqC v0.11.8 (https://www.bioinformatics.babraham.ac.uk/projects/fastqc/), and low-quality reads were removed with Trimmomatic v0.36 (Bolger, Lohse & Usadel, 2014) with a Phred cut-off value of Q = 30. To filter eukaryote contamination, we employed BWA-MEM v0.7 (Li & Durbin, 2010) with default parameters against the genome sequence of *Cathartes aura* (GCA_000699945.1) used as a proxy for *Vultur gryphus* since it was the closest known sequenced genome. Additionally, the genomic sequence of *Homo sapiens* (GCF_000001405.40), *Bos taurus* (GCF_002263795.2), *Equus caballus* (GCF_002863925.1), *Oryctolagus cuniculus* (GCA_000003625.1), *Gallus gallus* (GCF_000002315.6), *Felis catus* (GCA_000181335.4), *Sus scrofa* (GCA_000003025.6), *Rattus norvegicus* (GCF_000001895.5) and *Canis lupus familiaris* (GCA_000002285.2), downloaded from Genome database of NCBI (NCBI Resource Coordinators, 2018) (https://www.ncbi.nlm.nih.gov/) and used to filter the reads. These genomic sequences were chosen due to their usage to feed the birds kept at the CRAR or any noise introduced by human manipulation of the samples. Unmapped reads were maintained for further analysis.

The taxonomy assignment was performed using all reads that passed the quality and diet contamination filters with Kraken2 (Wood, Lu & Langmead, 2019) and MetaPhlAn v2.0 (Segata et al., 2012) with default parameters, employing code and database versions as were available in October 2019. All filtered reads were assembled with IDBA-UD v1.1.3 (Peng et al., 2012) using an iterative k-mer approach for contig assembly. After the taxonomy assignment, we used the top two most abundant species to perform a genome reference-guided assembly using MetaCompass (Cepeda et al., 2017).

### Functional profiling of Andean condor metagenome

We performed a gene prediction using Prodigal (Hyatt et al., 2010) for metagenomes with all other parameters set to their default values. Obtained amino acid sequences for each predicted gene were functionally annotated following an automated in-house pipeline (Isla et al., 2021). This pipeline employs Eggnog-mapper v2.0.1-14-gbf04860 (Huerta-Cepas et al., 2017) based on eggNOG 4.5 (Huerta-Cepas et al., 2016) using default parameters and Diamond v0.9.30.131 (Buchfink, Xie & Huson, 2015) for orthologous search, and COG (Clusters of Orthologous Genes) functional assignment, additionally we used InterproScan v5.31-70.0 (Jones et al., 2014) to detect homology based on protein domains. The metabolic pathways were reconstructed using KEGG mapper software (Kanehisa & Sato, 2020).

### Computational characterization of antibiotic resistance genes and virulence factors from the Andean condor gut microbiome

Drug resistance genes were determined by sequence similarity between translated genes in the metagenome and reported resistance genes using the sequence of The Comprehensive Antibiotic Resistance Database (CARD) v3.0.8 (card.mcmaster.ca) (Alcock et al., 2020) following the methodology of Cao and cols. Cao et al. (2020), who used at least 80% of sequence identity and e-value of 1e−10 as a cut-off to ensure the accuracy of antibiotic resistance gene detection.

Virulence factors genes were identified by sequence similarity between translated genes in the metagenome and known virulence factors from the Virulence Factor Database (VFDB) v5 (mgc.ac.cn/VFs) (Chen et al., 2005; Liu et al., 2019) as a reference, considering at least 60% sequence identity and e-value of 1e−10 (Escudeiro et al., 2019).

### Data availability

The metagenome raw reads obtained from the sequencing of the gut microbiome of the Andean condor were deposited into Sequence Read Archive (SRA) project SRR15043329.

## Results

### Metagenome sequencing and assembly

We sequenced for the first time the fecal metagenome of the Andean condor (*Vultur gryphus*). A total of 214,319,420 raw paired-end reads were generated by the HiSeq 2500 platform × 100 read length. After trimming, we conserved 51,415,866 high-quality reads. These reads were filtered to remove all sequences corresponding to diet or sample manipulation, the 28,761,042 reads were assembled, and we obtained 83,567 contigs (for details, see Table 1).

**Table 1:** Stats from scaffolds and predicted genes of Andean condor gut microbiome.

Number of contigs	83,657
Shortest contig length (nucleotides)	91
Largest contig length (nucleotides)	326,950
N50	1,829
N90	391
Mean length contig	1,036
Average GC content by contig (%)	38.16
Number of predicted genes in microbiome	142,429
Shortest gene	60
Largest gene	15,789
Average GC content by gene (%)	37.25

### The taxonomic composition of the Andean condor gut microbiome reveals a predominance of *Clostridium perfringens*

**Figure 1:**
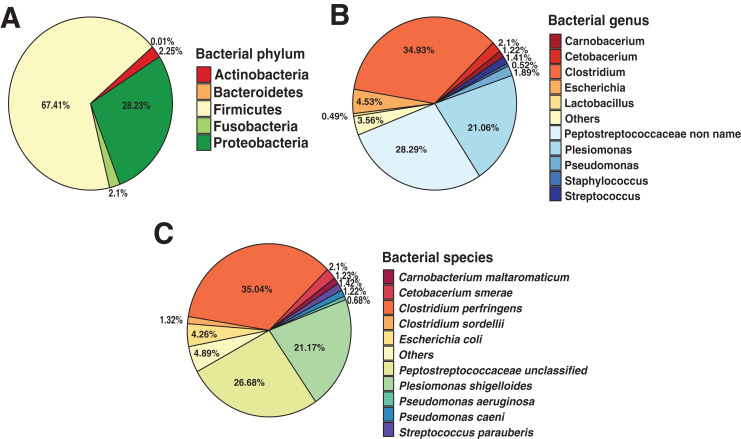
Andean condor (*Vultur gryphus*) gut microbiome composition. (A) Phylum level. (B) Genus level and (C) species level.

To identify the composition of the Andean condor gut microbiome, we performed a taxonomic assignment with all filtered reads. Our results showed 99.91% of reads corresponded to Bacteria, 0.8% to Viruses (Retroviridae), and 0.01% to Fungi (Ascomycota). Within the bacteria, the most abundant phylum was Firmicutes with a relative abundance of 67.41%, followed by Proteobacteria (28.23%), Actinobacteria (2.25%), Fusobacteria (2.1%) and Bacteroidetes (0.01%), shown in Fig. 1A. Further, we identified 38 bacterial genera, of which, *Clostridium* was the most abundant (34.93%) (Fig. 1B and Data S1), followed by unclassified *Peptostreptococcaceae* bacteria (28.29%) and *Plesiomonas* (21.06%).

These three genera compose 84.22% of the total bacterial abundance found in the Andean condor gut microbiome. Finally, 63 species were identified at the species level with *Clostridium perfringens*, an unclassified *Peptostreptococcaceae*, and *Plesiomonas shigelloides* representing 84.34% of the total abundance. Figure 1C and Data S1 show the relative abundance at the species level of the gut microbiome of the Andean condor.

### Functional analysis indicates a potential reservoir of antibiotic resistance genes and virulence factors in the Andean condor gut microbiome

**Figure 2:**
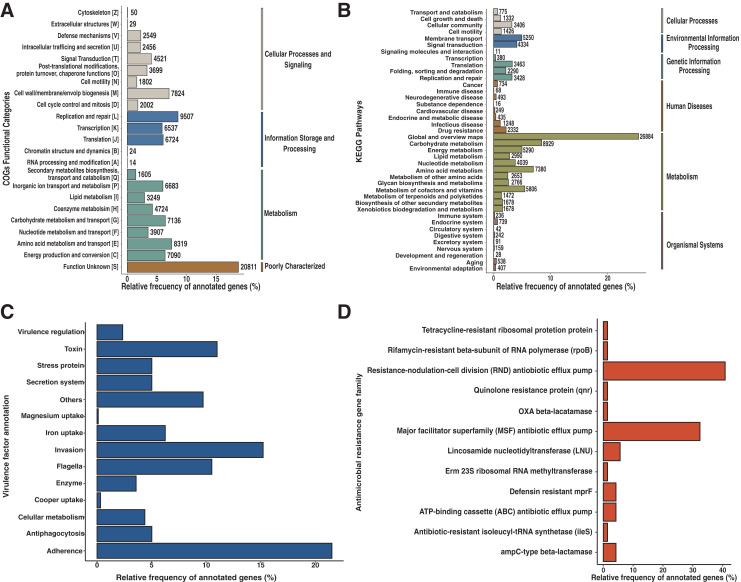
Functional characterization of Andean condor gut microbiome. (A) COG (Clusters of Orthologous Genes) categories level. (B) KEGG pathways. (C) Virulome, virulence factors metafunctions. (D) Antimicrobial resistance (AMR) genes families.

An entire repertory of 142,429 genes was predicted and functionally annotated from the Andean condor gut microbiome (Table 2). WeFrom obtained a functional assignment for 124,107 predicted genes (Table 2, Data S2). To evaluate the general features of annotated genes, we evaluate the abundance of COG functional categories (Fig. 2A). COG category S was the most abundant (18.7%) in our annotated predicted genes. This category corresponds to an unknown function. On the other hand, categories involved in replication and repair (L) (8.5%), amino acid metabolism and transport (E) (7.5%), cell wall biogenesis (M) (7%), carbohydrate and metabolism transport (G) (6.4%) and energy production and conservation (C) (6.37%) are the top five most identified genes.

**Table 2:** Andean condor gut microbiome gene functional annotation.

Annotated genes by kingdom	
Bacterial genes	110,052
Viral genes	1,085
Archaeal genes	53
Eukaryotic genes	63
Non taxonomic assignation genes	12,854
Non annotated genes	18,322

Based on our annotation protocol we identified 77,384 genes with at least one KO (KEEG ortholog) number. We used these KOs and made a pathway reconstruction. Our reconstruction was classified into six groups comprising 40 functional categories (Fig. 2B). We found that metabolism was the largest represented group with 67.7% of total annotated KO, with the most represented annotations among the metabolism of carbohydrates, amino acids, cofactors, and vitamins. The second most abundant functional group was environmental information processing, with 9.08% of annotated KO from the Andean condor gut microbiome. From this, “Membrane transport” was the most abundant category, followed by “Signal transduction” and “Signaling molecules and interaction”. The third most ample functional group was “Genetic information processing” (9.05%). The fourth group of most frequent KEGG annotations was “Cellular Processes and Signaling”, where cellular community and cellular motility were the most dominant categories. The remaining two groups of annotations were “Organismal system” and “Human Diseases and Organismal systems”, where “Drug resistance”, “Infectious disease” and “Cancers” were the most important categories within the group of Human diseases, which indicates the potential existence of a human pathogen reservoir in the microbiota of this animal.

Our results showed 1,786 genes annotated as virulence factors (VFs) in the Andean condor gut microbiome. Figure 2C and Data S3 present global functions for all VFs. We observed that functions involved in adherence, invasion, toxins, and flagella were most abundant in our gene set. Further examination of specific virulence features revealed capsule, polar flagella, lipooligosaccharide (LOS), elongation factor-Tu, polysaccharide capsule, flagella, Type IV pili, heme biosynthesis, flagellin subunit, fibronectin-binding protein, were the most abundant VFs in our sample.

Our homology search analysis detected 71 proteins related to 43 different ARGs families (Fig. 2D, Data S4), representing 11 different antibiotic classes and five classical mechanisms of antibiotic resistance. The most abundant ARGs, acting mainly through antibiotic efflux pumps, included three different superfamilies: ATP-binding cassette (ABC), major facilitator superfamily (MFS), and resistance-nodulation-cell division (RND) (Fig. 2D). Indeed, CRP (cyclic AMP receptor protein), a regulator that modulates multidrug resistance (Nishino, Senda & Yamaguchi, 2008) and *tetA* genes represented 16.9% of all AMR proteins found. A total of 22.53% of ARGs were related to antibiotic inactivation and target alteration, such as *AmpC-*type beta-lactamase, lincosamide nucleotidyltransferase (LNU), or defensin resistant (*mprF*). Regarding the antibiotics resistance classes, most of these genes are related to resistance against chemotherapeutic drugs such as fluoroquinolones (23.94%), tetracyclines (19.72%), and bacteriolytic beta-lactams antibiotics (19.72%). Less frequently, there are ARGs encoding for resistance to aminocoumarin (9.86%), lincosamide (7.04%), nucleoside antibiotic (5.63%), peptide antibiotic (5.63%), macrolide (5.6%), nitroimidazole (5.6%), mupirocin (1.41%) and rifamycin (1.41%).

### *Clostridium perfringens* and *Plesiomonas shigelloides* represent the most abundant species, in the gut microbiome of the Andean condor

**Figure 3:**
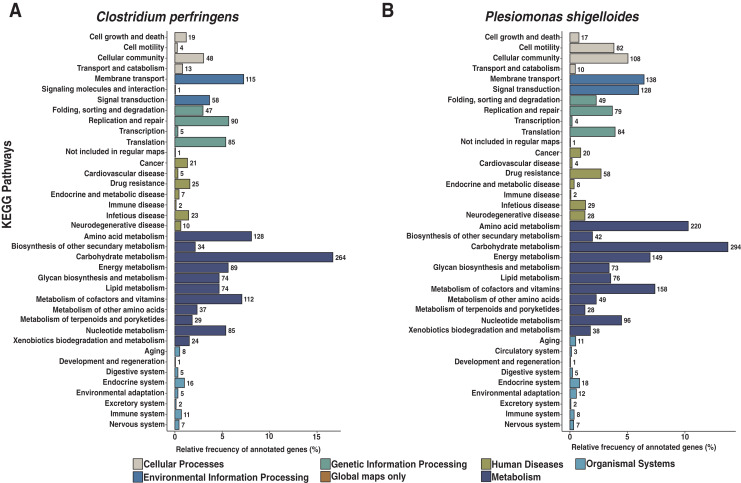
Functional characterization of KEGG pathways level of *C. perfringens* and *P. shigelloides* genome assemblies. (A) AEGG pathways annotation *C. perfringens**. ***(B) KEGG pathways annotation *P. shigelloides*.

**Figure 4:**
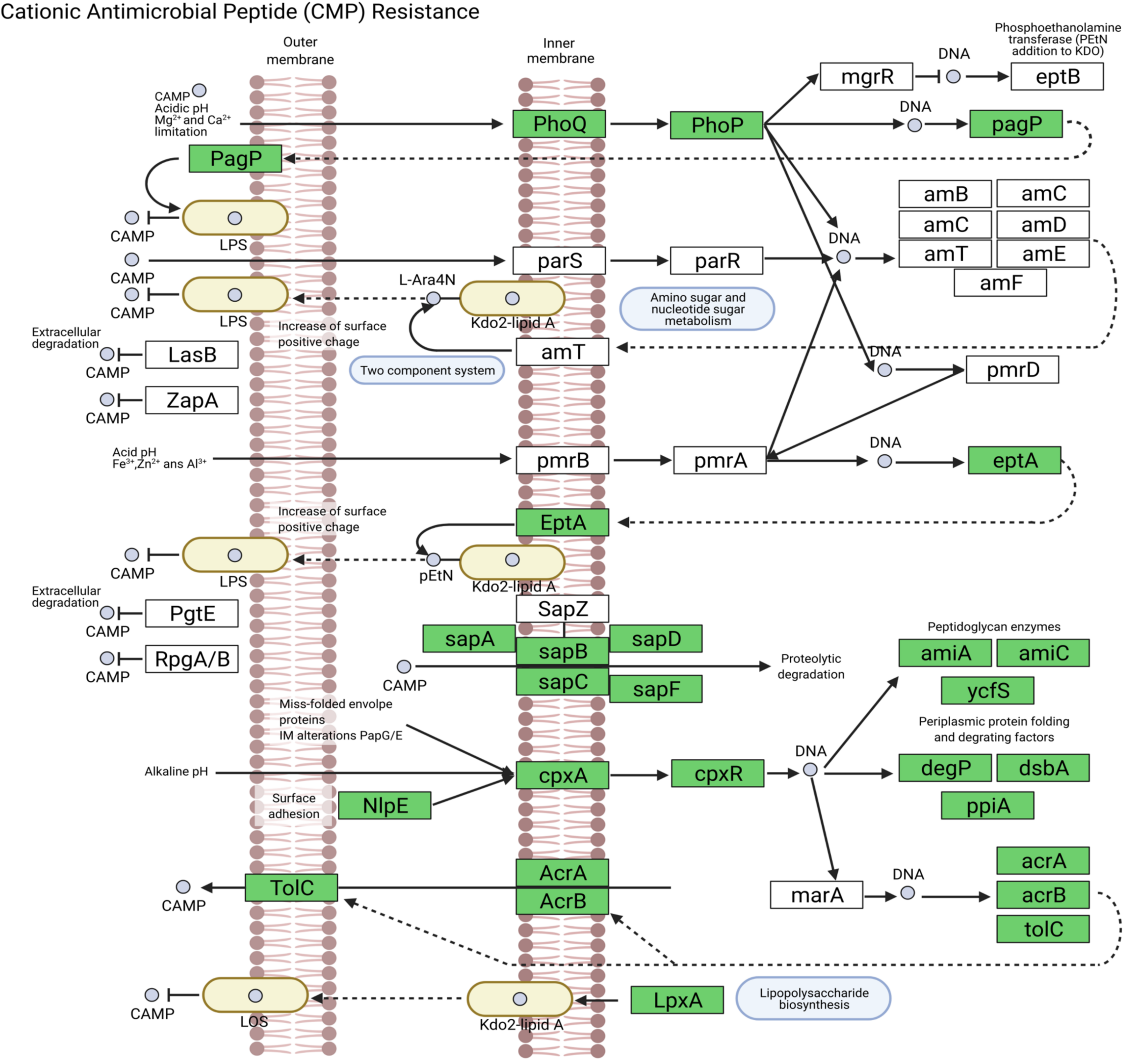
Cationic antimicrobial peptide (CAMP) resistance pathway in *P. shigelloides*.

We assembled all reads corresponding to the top two species more frequent in the condor gut microbiome. These reads were aligned against publicly available genomes of *Clostridium perfringens* (accession GCF_020138775.1) and *Plesiomonas shigelloides* (accession GCF_020991025.1) to estimate the completeness of the assemblies generated from our data. Interestingly, we found between 94% to 98% of completeness for *C. perfringens* and *P. shigelloides* (Table 3), even if these genomes assembled from the metagenome are likely to belong to different strains than those found in public databases and the differences can arise because of this (Yang et al., 2021). For *C. perfringens* we identified 2,947 genes, of which 2,792 have a functional annotation (Fig. 3A, Data S5). Our analysis revealed 25 genes that act in pathways related to antibiotic resistance, such as beta-lactam and vancomycin resistance. We identify the presence of the regulatory section (*vanR* and *vanS*), and the accessory gene *vanY* of van operon, but the resistance genes of van operon were not identified by our analyses, other genes such *murF*, *murG*, *mraY*, *ddl*, and *alr* also related to vancomycin resistance were distinguished. However, the complete pathway of vancomycin resistance in *C. perfringens* could not be mapped; the function of this set of genes is summarized in Data S5. On the other hand, for *P. shigelloides* we obtain an annotation for 3,036 genes (Fig. 3B, Data S6), in KEGG pathways annotation we observed the presence of 52 genes playing a role in different drug resistance pathways (Data S6), with our search, we computationally characterize an entry pathway for Cationic antimicrobial peptide (CAMP) resistance (Fig. 4) which is not present in *C. perfringens*.

**Table 3:** Stats for *Clostridium perfringens* and *Plesiomonas shigelloides* genome assemblies.

Specie	*Clostridium perfringens*	*Plesiomonas shigelloides*
Contigs	149	454
N50	390,285	66,283
N90	63,986	12,799
Completeness (%)	98.34	93.94
Predicted genes	2,947	3,296
Annotated genes	2,792	3,096

## Discussion

Microbial diversity and richness in an organism correlate strongly with habitat, lifestyle, and diet, among other factors (Boon et al., 2014). This information is also relevant for preserving biodiversity and pathogens surveillance in terms of potential zoonotic issues. Another issue regarding the microbial diversity of the Andean condor gut is its geographical distribution, with different areas often associated with different food sources (Sun et al., 2022). This species is found along the Andes in Colombia, Ecuador, Peru, Chile, Bolivia, and western Argentina to Tierra del Fuego and often travels more than 200 km a day in search of carrion (Naveda-Rodríguez et al., 2016; Pérez-García et al., 2018; BirdLife International, 2020).

Andean condor is essential for ecosystem health and functioning (sanitation service). Thus, the Andean condor diet is also an additional parameter related to the relative abundance patterns of microorganisms that inhabit mainly the gut. The scavenger habits are also a high source of microorganism diversity since they feed on the largest carcasses available which can include a broad spectrum of animals such as llamas (*Lama glama*), alpacas (*Vicugna pacos*), guanacos (*Lama guanicoe*) and armadillos (Lambertucci et al., 2009). However, most condors can live largely off of domestic animals more widespread in South America, such as cattle, horses, donkeys, sheep, pigs, goats, and dogs (Encyclopedia of Life, 2018; BirdLife International, 2020; Duclos et al., 2020).

According to its lifestyle as the largest scavenger bird in South America, the Andean condor is a unique species for studying wildlife pathogens and its role as a reservoir of potential pathogens that threaten animal and human health in this area of the world (Wiemeyer et al., 2021). Therefore, the study of the Andean condor gut microbiota will contribute to the understanding of the emergence of novel pathogen agents and the interactions among animals, wildlife and human populations living in proximity. Thus, understanding the status of wildlife species as hosts for pathogens may play a relevant during this time of anthropogenic environmental change (Becker, Streicker & Altizer, 2015; Llanos-Soto & González-Acuña, 2019).

The microbiome we studied here is from captive condors kept at the CRAR rehabilitation center, making this artificial habitat a possible source for bias in the composition of the gut microbiome. However, Roggenbuck et al. (2014) reported that gut microbiomes from captive and wild sampled vultures are remarkably similar. However, this conservation has been more recently questioned (Becker et al., 2020; Dallas & Warne, 2022). In addition, other works (Meng et al., 2017; Zepeda Mendoza et al., 2018) reported the existence of pathogens in the vulture’s microbiome, an indication of adaptation processes in the vultures, and a possible protective role from pathogenic microbes. Nonetheless, our study is the first reported characterization of the microbial diversity of the gut microbiome of the Andean condor, and even if the analyzed sample is from captive animals. It is also important to consider that birds at the CRAR rehabilitation center are fed with donated carcasses of farmed animals, a diet that has been reported to be highly similar to those of wild birds.

According to our results, the Andean condor gut microbiome is dominated by two major phyla of bacteria. These two phyla, Firmicutes and Proteobacteria, comprise 95.6% of the bacteria. Clostridia species are part of the Firmicutes phylum. These species have been documented as the cause of severe food poisoning in humans and chickens and are responsible for the periodic deaths of wild birds (Roggenbuck et al., 2014). Domestic and wild birds have a high relative abundance of Proteobacteria (Grond et al., 2018). Our functional annotation of the metagenome shows that the Andean condor is a host that tolerates the bacteria and their toxins since virulence factors were found in physically damaged animals that were healthy otherwise. Moreover, this microbial biodiversity contributes to obtaining nutrients provided by bacterial degradation of carrion. Additionally, we identify in our sample two different *Brachybacterium* species, *Brachybacterium faecium* and a *B. unclassified* strain, which agrees with previous findings in other vultures described by Tak et al. (2018) (Data S1). These species had been isolated from wide-ranging sources demonstrating that they have adapted to diverse environmental conditions (Tak et al., 2018). Efflux pumps and AMR coding genes, the most abundant protein types coded by genes found in our analysis (Fig. 2D), are recognized as relevant mechanisms to explain multiple antibiotics in multidrug-resistant bacteria (Cao et al., 2020). Thus, antibiotic target protection and antibiotic inactivation were the most enriched annotations in the genes of the Andean condor metagenome. For example, drug efflux mechanisms are represented in the RND, MFS gene families, both part of AMR genes. AMR gene families also have been enriched in the Californian condor microbiomes (Jacobs et al., 2019). Another interesting AMR gene family found in the Andean condor microbiome was the B-lactamase gene. Extended-spectrum B-lactamase genes (ESBL)-producing Enterobacterales are now classified as critical pathogens by the World Health Organization (Shrivastava, Shrivastava & Ramasamy, 2018; Tacconelli et al., 2018a, 2018b) and according to our diversity results enterobacteriales is part of one of the most abundant phyla in the Andean condor microbiome: Proteobacteria (Data S4). Importantly, our findings agree with previous results reported with sample culturing followed by mass spectrometry identification of *E. coli* carried AMR genes in Andean condor (Fuentes-Castillo et al., 2020), reinforcing in this way, the relevance of our microbiome study.

*Plesiomonas shigelloides* was previously described as part of the microbiome of wild vultures such as the turkey vulture (*Cathartes aura*) (Winsor, Bloebaum & Mathewson, 1981). Our results demonstrated the predominance of this specie in the Andean condor intestinal microbiome. Additionally, our gene annotation found several protein-coding genes related to the cationic antimicrobial peptides (CAMPs) resistance (Fig. 4). CAMPs are a wide group with microbicidal properties (Band & Weiss, 2015). CAMPs generally destabilize bacterial membranes by interacting with anionic head groups and hydrophobic fatty acid chains (Wimley, 2010). In a recent study, Yin et al. (2020) described diverse multidrug resistance mechanisms in *P. shigelloides* such CAMPs resistance, acquired by horizontal gene transfer. Another study also reveals the potential of *P. shigelloides* as a reservoir for antimicrobial resistance in aquaculture farms (Martins et al., 2019). This is concordant with the findings of Blanco et al. (2020) and Zepeda Mendoza et al. (2018) who described that Old and New World vultures act as reservoirs for bacteria that may be pathogenic to other animals which are carried of genes involved in the resistance to antimicrobial agents in the gut microbiome (Roggenbuck et al., 2014).

Genes contributing to toxin production, colonization, and evasion of the host immune response are functionally relevant in the Andean microbiomes. These genes may indicate an adaptation of Andean condors to the bacteria in their gut microbiome. This statement makes sense considering our results since adherence, invasion and toxin were the most abundant virulence factors in the Andean condor gut microbiome. These virulence factors can cause diseases in humans and animals and are part of different classes such as proteases, capsule proteins, ureases, and fibronectins (Data S3).

## Conclusion

Metagenomics surveillance of wildlife could help to know about the microbiota dynamics, track pathogens across natural and captive populations, make available reference genomes of microorganisms, and improve understanding of the epidemiology and biology of microorganisms in this unexplored biome. The knowledge will lead to potential intervention in different diseases caused by bacteria to potentially translate surveillance outcomes into tools for pathogens control and wildlife conservation. In this way, the metagenomics analysis performed in this work highlights the ability of the Andean condor to act as an environmental reservoir and potential vector for critical priority pathogens to other animals, such as *C. perfringens* and *P. shigelloides* that contain relevant genetics elements associated with adaptation processes such as AMR genes and virulence factors.

## Supplemental Information

10.7717/peerj.15235/supp-1 Supplemental Information 1 Genus, species and relative abundance identified in gut microbiome from Andean Condor.

10.7717/peerj.15235/supp-2 Supplemental Information 2 Metagenomics analysis of gut microbiome from Andean Condor.

10.7717/peerj.15235/supp-3 Supplemental Information 3 Gene identification.

10.7717/peerj.15235/supp-4 Supplemental Information 4 AMR Annotation, metafunction and mechanisms.

10.7717/peerj.15235/supp-5 Supplemental Information 5 Gene annotation of C. prefringens genome assembled from Andean Condor metagenome.

10.7717/peerj.15235/supp-6 Supplemental Information 6 Gene annotation of P. shigeloides genome assembled from Andean Condor metagenome.

## Additional Information and Declarations

### Data Availability

The following information was supplied regarding data availability: The metagenome raw reads obtained from the sequencing of gut microbiome of Andean Condor is available at Sequence Read Archive (SRA): SRR15043329.

## References

[ref-1] Alcock BP, Raphenya AR, Lau TTY, Tsang KK, Bouchard M, Edalatmand A, Huynh W, Nguyen ALV, Cheng AA, Liu S, Min SY, Miroshnichenko A, Tran HK, Werfalli RE, Nasir JA, Oloni M, Speicher DJ, Florescu A, Singh B, Faltyn M, Hernandez-Koutoucheva A, Sharma AN, Bordeleau E, Pawlowski AC, Zubyk HL, Dooley D, Griffiths E, Maguire F, Winsor GL, Beiko RG, Brinkman FSL, Hsiao WWL, Domselaar GV, McArthur AG (2020). CARD 2020: antibiotic resistome surveillance with the comprehensive antibiotic resistance database. Nucleic Acids Research.

[ref-2] Band VI, Weiss DS (2015). Mechanisms of antimicrobial peptide resistance in gram-negative bacteria. Antibiotics.

[ref-3] Becker AAMJ, Harrison SWR, Whitehouse-Tedd G, Budd JA, Whitehouse-Tedd KM (2020). Integrating gut bacterial diversity and captive husbandry to optimize vulture conservation. Frontiers in Microbiology.

[ref-4] Becker DJ, Streicker DG, Altizer S (2015). Linking anthropogenic resources to wildlife-pathogen dynamics: a review and meta-analysis. Ecology Letters.

[ref-5] BirdLife International (2020). Vultur gryphus. https://www.iucnredlist.org/species/22697641/181325230.

[ref-6] Blanco G, López-Hernández I, Morinha F, López-Cerero L (2020). Intensive farming as a source of bacterial resistance to antimicrobial agents in sedentary and migratory vultures: implications for local and transboundary spread. The Science of the Total Environment.

[ref-7] Bolger AM, Lohse M, Usadel B (2014). Trimmomatic: a flexible trimmer for Illumina sequence data. Bioinformatics.

[ref-8] Boon E, Meehan CJ, Whidden C, Wong DHJ, Langille MGI, Beiko RG (2014). Interactions in the microbiome: communities of organisms and communities of genes. FEMS Microbiology Reviews.

[ref-9] Buchfink B, Xie C, Huson DH (2015). Fast and sensitive protein alignment using DIAMOND. Nature Methods.

[ref-10] Buechley ER, Şekercioğlu ÇH (2016). The avian scavenger crisis: looming extinctions, trophic cascades, and loss of critical ecosystem functions. Biological Conservation.

[ref-11] Cao J, Hu Y, Liu F, Wang Y, Bi Y, Lv N, Li J, Zhu B, Gao GF (2020). Metagenomic analysis reveals the microbiome and resistome in migratory birds. Microbiome.

[ref-12] Cepeda V, Liu B, Almeida M, Hill CM, Koren S, Treangen TJ, Pop M (2017). MetaCompass: reference-guided assembly of metagenomes. bioRxiv.

[ref-13] Chen L, Yang J, Yu J, Yao Z, Sun L, Shen Y, Jin Q (2005). VFDB: a reference database for bacterial virulence factors. Nucleic Acids Research.

[ref-14] Chung O, Jin S, Cho YS, Lim J, Kim H, Jho S, Kim HM, Jun J, Lee H, Chon A, Ko J, Edwards J, Weber JA, Han K, O’Brien SJ, Manica A, Bhak J, Paek WK (2015). The first whole genome and transcriptome of the cinereous vulture reveals adaptation in the gastric and immune defense systems and possible convergent evolution between the Old and New World vultures. Genome Biology.

[ref-15] Dallas JW, Warne RW (2022). Captivity and animal microbiomes: potential roles of microbiota for influencing animal conservation. Microbial Ecology.

[ref-16] Duclos M, Sabat P, Newsome SD, Pavez EF, Galbán-Malagón C, Jaksic FM, Quirici V (2020). Latitudinal patterns in the diet of Andean condor (Vultur gryphus) in Chile: contrasting environments influencing feeding behavior. Science of the Total Environment.

[ref-17] Encyclopedia of Life (2018). Andean condor. https://eol.org/pages/1049160/articles.

[ref-18] Escudeiro P, Pothier J, Dionisio F, Nogueira T (2019). Antibiotic resistance gene diversity and virulence gene diversity are correlated in human gut and environmental microbiomes. mSphere.

[ref-19] Fuentes-Castillo D, Esposito F, Cardoso B, Dalazen G, Moura Q, Fuga B, Fontana H, Cerdeira L, Dropa M, Rottmann J, González-Acuña D, Catão-Dias JL, Lincopan N (2020). Genomic data reveal international lineages of critical priority Escherichia coli harbouring wide resistome in Andean condors (Vultur gryphus Linnaeus, 1758). Molecular Ecology.

[ref-20] Gaengler H, Clum N (2015). Investigating the impact of large carcass feeding on the behavior of captive Andean condors (Vultur gryphus) and its perception by zoo visitors. Zoo Biology.

[ref-21] García-Alfonso M, Morales-Reyes Z, Gangoso L, Bouten W, Sánchez-Zapata JA, Serrano D, Donázar JA (2019). Probing into farmers’ perceptions of a globally endangered ecosystem service provider. Ambio.

[ref-22] Gilbert JA, Dupont CL (2011). Microbial metagenomics: beyond the genome. Annual Review of Marine Science.

[ref-23] Grond K, Sandercock BK, Jumpponen A, Zeglin LH (2018). The avian gut microbiota: community, physiology and function in wild birds. Journal of Avian Biology.

[ref-24] Huerta-Cepas J, Forslund K, Coelho LP, Szklarczyk D, Jensen LJ, von Mering C, Bork P (2017). Fast genome-wide functional annotation through orthology assignment by eggNOG-Mapper. Molecular Biology and Evolution.

[ref-25] Huerta-Cepas J, Szklarczyk D, Forslund K, Cook H, Heller D, Walter MC, Rattei T, Mende DR, Sunagawa S, Kuhn M, Jensen LJ, von Mering C, Bork P (2016). eggNOG 4.5: a hierarchical orthology framework with improved functional annotations for eukaryotic, prokaryotic and viral sequences. Nucleic Acids Research.

[ref-26] Hyatt D, Chen GL, Locascio PF, Land ML, Larimer FW, Hauser LJ (2010). Prodigal: prokaryotic gene recognition and translation initiation site identification. BMC Bioinformatics.

[ref-27] Isla A, Eduardo Martinez-Hernandez J, Levipan HA, Haussmann D, Figueroa J, Rauch MC, Maracaja-Coutinho V, Yañez A (2021). Development of a multiplex PCR assay for genotyping the fish pathogen piscirickettsia salmonis through comparative genomics. Frontiers in Microbiology.

[ref-65] IUCN (2022). The IUCN red list of threatened species. https://www.iucnredlist.org.

[ref-28] Jacobs L, McMahon BH, Berendzen J, Longmire J, Gleasner C, Hengartner NW, Vuyisich M, Cohn JR, Jenkins M, Bartlow AW, Fair JM (2019). California condor microbiomes: bacterial variety and functional properties in captive-bred individuals. PLOS ONE.

[ref-29] Johnson JA, Brown JW, Fuchs J, Mindell DP (2016). Multi-locus phylogenetic inference among New World Vultures (Aves: Cathartidae). Molecular Phylogenetics and Evolution.

[ref-30] Jones P, Binns D, Chang HY, Fraser M, Li W, McAnulla C, McWilliam H, Maslen J, Mitchell A, Nuka G, Pesseat S, Quinn AF, Sangrador-Vegas A, Scheremetjew M, Yong SY, Lopez R, Hunter S (2014). InterProScan 5: genome-scale protein function classification. Bioinformatics.

[ref-31] Kanehisa M, Sato Y (2020). KEGG mapper for inferring cellular functions from protein sequences. Protein Science: A Publication of the Protein Society.

[ref-32] Lambertucci SA, Navarro J, Sanchez Zapata JA, Hobson KA, Alarcón PAE, Wiemeyer G, Blanco G, Hiraldo F, Donázar JA (2018). Tracking data and retrospective analyses of diet reveal the consequences of loss of marine subsidies for an obligate scavenger, the Andean condor. Proceedings of the Royal Society B: Biological Sciences.

[ref-33] Lambertucci SA, Trejo A, Di Martino S, Sánchez-Zapata JA, Donázar JA, Hiraldo F (2009). Spatial and temporal patterns in the diet of the Andean condor: ecological replacement of native fauna by exotic species. Animal Conservation.

[ref-66] Laxminarayan R, Duse A, Wattal C, Zaidi AK, Wertheim HF, Sumpradit N, Vlieghe E, Hara GL, Gould IM, Goossens H, Greko C, So AD, Bigdeli M, Tomson G, Woodhouse W, Ombaka E, Peralta AQ, Qamar FN, Mir F, Kariuki S, Bhutta ZA, Coates A, Bergstrom R, Wright GD, Brown ED, Cars O (2013). Antibiotic resistance-the need for global solutions. The Lancet Infectious Diseases.

[ref-34] Li H, Durbin R (2010). Fast and accurate long-read alignment with Burrows–Wheeler transform. Bioinformatics.

[ref-35] Liu B, Zheng D, Jin Q, Chen L, Yang J (2019). VFDB 2019: a comparative pathogenomic platform with an interactive web interface. Nucleic Acids Research.

[ref-36] Llanos-Soto S, González-Acuña D (2019). Knowledge about bacterial and viral pathogens present in wild mammals in Chile: a systematic review. Revista Chilena de Infectologia: Organo Oficial de la Sociedad Chilena de Infectologia.

[ref-37] Martins AFM, Pinheiro TL, Imperatori A, Freire SM, Sá-Freire L, Moreira BM, Bonelli RR (2019). Plesiomonas shigelloides: a notable carrier of acquired antimicrobial resistance in small aquaculture farms. Aquaculture.

[ref-38] Martín R, Miquel S, Langella P, Bermúdez-Humarán LG (2014). The role of metagenomics in understanding the human microbiome in health and disease. Virulence.

[ref-39] Mendes LW, Braga LPP, Navarrete AA, de Souza DG, Silva GGZ, Tsai SM (2017). Using metagenomics to connect microbial community biodiversity and functions. Current Issues in Molecular Biology.

[ref-41] Méndez D, Olea PP, Sarasola JH, Vargas FH, Astore V, Escobar-Gimpel V, Estrada-Pacheco R, Gordillo S, Jácome NL, Kohn-Andrade S, Kusch A, Naveda-Rodríguez A, Narváez F, Parrado-Vargas MA, Piana RP, Restrepo-Cardona JS, Wallace RB (2021). Vulnerable Andean condors in steep decline. Science.

[ref-42] Méndez DR, Soria-Auza RW, Hernán Vargas F, Herzog SK (2015). Population status of Andean condors in central and southern Bolivia. Journal of Field Ornithology.

[ref-40] Meng X, Lu S, Yang J, Jin D, Wang X, Bai X, Wen Y, Wang Y, Niu L, Ye C, Rosselló-Móra R, Xu J (2017). Metataxonomics reveal vultures as a reservoir for Clostridium perfringens. Emerging Microbes & Infections.

[ref-43] Naveda-Rodríguez A, Vargas FH, Kohn S, Zapata-Ríos G (2016). Andean condor (Vultur gryphus) in Ecuador: geographic distribution, population size and extinction risk. PLOS ONE.

[ref-44] NCBI Resource Coordinators (2018). Database resources of the national center for biotechnology information. Nucleic Acids Research.

[ref-45] Nishino K, Senda Y, Yamaguchi A (2008). CRP regulator modulates multidrug resistance of Escherichia coli by repressing the mdtEF multidrug efflux genes. The Journal of Antibiotics.

[ref-46] Ogada DL, Keesing F, Virani MZ (2012). Dropping dead: causes and consequences of vulture population declines worldwide. Annals of the New York Academy of Sciences.

[ref-47] Peng Y, Leung HCM, Yiu SM, Chin FYL (2012). IDBA-UD: a de novo assembler for single-cell and metagenomic sequencing data with highly uneven depth. Bioinformatics.

[ref-48] Pérez-García JM, Sánchez-Zapata JA, Lambertucci SA, Hiraldo F, Donázar JA (2018). Low-frequency, threatened habitats drive the large-scale distribution of Andean Condors in southern Patagonia. Ibis.

[ref-49] Roggenbuck M, Bærholm Schnell I, Blom N, Bælum J, Bertelsen MF, Sicheritz-Pontén T, Sørensen SJ, Gilbert MTP, Graves GR, Hansen LH (2014). The microbiome of new world vultures. Nature Communications.

[ref-50] Segata N, Waldron L, Ballarini A, Narasimhan V, Jousson O, Huttenhower C (2012). Metagenomic microbial community profiling using unique clade-specific marker genes. Nature Methods.

[ref-51] Seibold I, Helbig AJ (1995). Evolutionary history of new and old world vultures inferred from nucleotide sequences of the mitochondrial cytochrome b gene. Philosophical Transactions of the Royal Society of London. Series B, Biological Sciences.

[ref-52] Shrivastava S, Shrivastava P, Ramasamy J (2018). World health organization releases global priority list of antibiotic-resistant bacteria to guide research, discovery, and development of new antibiotics. Journal of Medical Society.

[ref-53] Sun F, Chen J, Liu K, Tang M, Yang Y (2022). The avian gut microbiota: diversity, influencing factors, and future directions. Frontiers in Microbiology.

[ref-54] Tacconelli E, Carrara E, Savoldi A, Harbarth S, Mendelson M, Monnet DL, Pulcini C, Kahlmeter G, Kluytmans J, Carmeli Y, Ouellette M, Outterson K, Patel J, Cavaleri M, Cox EM, Houchens CR, Grayson ML, Hansen P, Singh N, Theuretzbacher U, Magrini N, WHO Pathogens Priority List Working Group (2018a). Discovery, research, and development of new antibiotics: the WHO priority list of antibiotic-resistant bacteria and tuberculosis. The Lancet Infectious Diseases.

[ref-55] Tacconelli E, Sifakis F, Harbarth S, Schrijver R, van Mourik M, Voss A, Sharland M, Rajendran NB, Rodríguez-Baño J, Bielicki J, de Kraker M, Gandra S, Gastmeier P, Gilchrist K, Gikas A, Gladstone BP, Goossens H, Jafri H, Kahlmeter G, Leus F, Luxemburger C, Malhotra-Kumar S, Marasca G, McCarthy M, Navarro MD, Nuñez-Nuñez M, Oualim A, Price J, Robert J, Sommer H, von Cube M, Vuong C, Wiegand I, Witschi AT, Wolkewitz M (2018b). Surveillance for control of antimicrobial resistance. The Lancet Infectious Diseases.

[ref-56] Tak EJ, Kim PS, Hyun DW, Kim HS, Lee JY, Kang W, Sung H, Shin NR, Kim MS, Whon TW, Bae JW (2018). Phenotypic and genomic properties of brachybacterium vulturis sp. nov. and brachybacterium avium sp. nov. Frontiers in Microbiology.

[ref-57] Wiemeyer GM, Plaza PI, Bustos CP, Muñoz AJ, Lambertucci SA (2021). Exposure to anthropogenic areas may influence colonization by zoonotic microorganisms in scavenging birds. International Journal of Environmental Research and Public Health.

[ref-58] Wimley WC (2010). Describing the mechanism of antimicrobial peptide action with the interfacial activity model. ACS Chemical Biology.

[ref-59] Wink M (1995). Phylogeny of old and new world vultures (Aves: Accipitridae and Cathartidae) inferred from nucleotide sequences of the mitochondrial cytochrome b gene. Zeitschrift fur Naturforschung. C, Journal of Biosciences.

[ref-60] Winsor DK, Bloebaum AP, Mathewson JJ (1981). Gram-negative, aerobic, enteric pathogens among intestinal microflora of wild turkey vultures (Cathartes aura) in west central Texas. Applied and Environmental Microbiology.

[ref-61] Wood DE, Lu J, Langmead B (2019). Improved metagenomic analysis with Kraken 2. Genome Biology.

[ref-62] Yang C, Chowdhury D, Zhang Z, Cheung WK, Lu A, Bian Z, Zhang L (2021). A review of computational tools for generating metagenome-assembled genomes from metagenomic sequencing data. Computational and Structural Biotechnology Journal.

[ref-63] Yin Z, Zhang S, Wei Y, Wang M, Ma S, Yang S, Wang J, Yuan C, Jiang L, Du Y (2020). Horizontal gene transfer clarifies taxonomic confusion and promotes the genetic diversity and pathogenicity of plesiomonas shigelloides. mSystems.

[ref-64] Zepeda Mendoza ML, Roggenbuck M, Manzano Vargas K, Hansen LH, Brunak S, Gilbert MTP, Sicheritz-Pontén T (2018). Protective role of the vulture facial skin and gut microbiomes aid adaptation to scavenging. Acta Veterinaria Scandinavica.

